# Bioactivity, Health Benefits, and Related Molecular Mechanisms of Curcumin: Current Progress, Challenges, and Perspectives

**DOI:** 10.3390/nu10101553

**Published:** 2018-10-19

**Authors:** Xiao-Yu Xu, Xiao Meng, Sha Li, Ren-You Gan, Ya Li, Hua-Bin Li

**Affiliations:** 1 Department of Nutrition, School of Public Health, Sun Yat-Sen University, Guangzhou 510080, China; xuxy53@mail2.sysu.edu.cn (X.-Y.X.); mengx7@mail2.sysu.edu.cn (X.M.); liya28@mail3.sysu.edu.cn (Y.L.); 2 School of Chinese Medicine, Li Ka Shing Faculty of Medicine, The University of Hong Kong, Hong Kong 999077, China; u3003781@connect.hku.hk; 3 Department of Food Science & Technology, School of Agriculture and Biology, Shanghai Jiao Tong University, Shanghai 200240, China; 4 South China Sea Bioresource Exploitation and Utilization Collaborative Innovation Center, Sun Yat-Sen University, Guangzhou 510006, China

**Keywords:** curcumin, gut metabolism, health benefits, antioxidant, anticancer, anti-inflammatory, molecular mechanisms

## Abstract

Curcumin is a principal curcuminoid of turmeric (*Curcuma longa*), which is commonly used as a spice in cooking and a yellow pigment in the food processing industry. Recent studies have demonstrated that curcumin has a variety of biological activities and pharmacological performances, providing protection and promotion of human health. In addition to presenting an overview of the gut metabolism of curcumin, this paper reviews the current research progress on its versatile bioactivity, such as antioxidant, anti-inflammatory, and immune-regulatory activities, and also intensively discusses its health benefits, including the protective or preventive effects on cancers and diabetes, as well as the liver, nervous system, and cardiovascular systems, highlighting the potential molecular mechanisms. Besides, the beneficial effects of curcumin on human are further stated based on clinical trials. Considering that there is still a debate on the beneficial effects of curcumin, we also discuss related challenges and prospects. Overall, curcumin is a promising ingredient of novel functional foods, with protective efficacy in preventing certain diseases. We hope this comprehensive and updated review will be helpful for promoting human-based studies to facilitate its use in human health and diseases in the future.

## 1. Introduction

**Figure 1:**
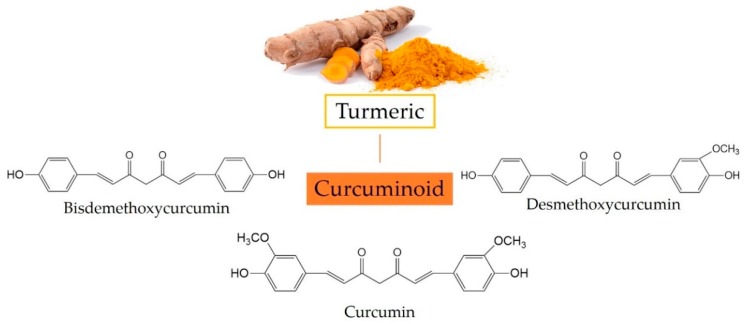
Three major curcuminoids in turmeric and their chemical structures.

Turmeric, the powdered rhizome of *Curcuma longa*, is a member of the Zingiberaceae family [1]. The extract of turmeric contains three major curcuminoids (Figure 1): Curcumin (60–70%), demethoxycurcumin (20–27%), and bisdemethoxycurcumin (10–15%) [2]. Curcumin ((1*E*,6*E*)-1,7-bis(4-hydroxy-3-methoxyphenyl)-1,6-heptadiene-3,5-dione) has two aromatic *O*-methoxy phenolic groups, a β-dicarbonyl moiety and a seven-carbon linker containing two enone moieties. Structural modifications with different functional groups have improved the physicochemical and bioactive properties of curcumin [3]. There have been numerous studies on the bioactivity and health benefits of curcumin, such as antioxidant, anti-inflammatory, immune regulatory, anticancer, antidiabetic, neuro-protective, cardiovascular protective, and hepatoprotective effects [4,5]. To better understand the beneficial effects of curcumin on health, we conducted a search for relevant articles in the Institute of Science Information (ISI) Web of Science core collection and PubMed from 2013 to the present. In this review, we first state the metabolic pathways of curcumin in the intestine, followed by summarizing its diverse bioactivity and health benefits, and also discuss related molecular mechanisms. Therefore, we hope this updated review can provide a better understanding of the beneficial effects of curcumin.

## 2. The Metabolism of Curcumin

**Figure 2:**
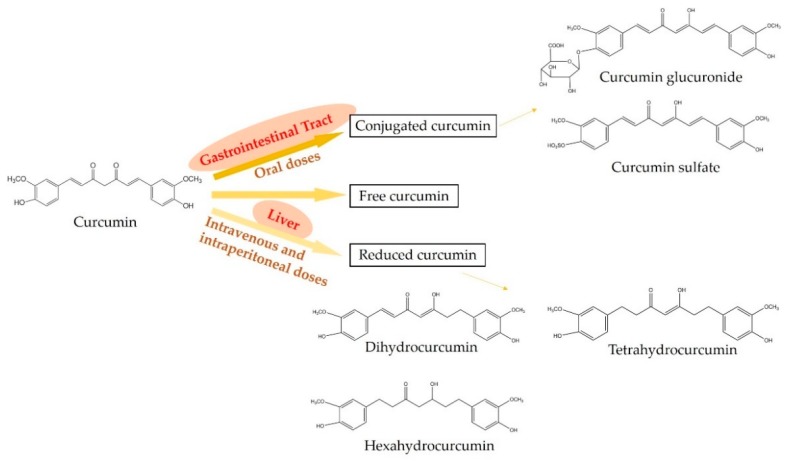
Metabolic pathways of curcumin. Oral administration mainly metabolizes curcumin into conjugated curcumin, while intravenous or intraperitoneal administration mainly leads to reduced curcumin. In addition, minor free and intact curcumin can be detected in plasma after any administration.

The metabolism of curcumin is critical for its potent biological activities as well as the beneficial effects on health [6]. In the mammalian body, curcumin can be presented in three major forms, which are free, conjugated, and reduced states [7,8,9] (Figure 2). Oral administration mainly metabolizes curcumin into the conjugated curcumin via glucuronidation and sulfation. Especially, the finding demonstrates that the gastrointestinal tract plays an important role in the glucuronidation of curcuminoids in human [10]. In addition, intravenous or intraperitoneal administration leads to the reduction of curcumin into dihydrocurcumin, tetrahydrocurcumin, and hexahydrocurcumin [11]. However, minor free and intact curcumin can be detected in plasma after the administration [12]. On the other hand, the metabolism of curcuminoids mainly leads to reductive metabolites, for example, hexahydrocurcuminoids are the major reductive metabolites observed in both male and female rat liver [13].

Due to the low chemical stability and poor bioavailability of curcumin, more attention has been paid to its metabolites [14]. Although the major structure of its metabolites is consistent with curcumin, the minor differences in the structure of metabolites improve their chemical stability. For example, the hexahydrocurcumin has the same phenolic groups or diketo moieties as curcumin, but has no olefinic double bonds, leading to hexahydrocurcumin being more stable than curcumin at a physiological pH of 7.4 [15]. In the intestine, the absorption of curcumin is poor, while the reductive and conjugated curcumin metabolites show moderate absorption [16]. Hence, it is hypothesized that the biological effects of curcumin in tissues, such as liver and kidney, may be attributed to the curcumin metabolites [17]. However, the metabolism or degradation of curcumin can affect the bioactivity of curcumin. A finding shows that curcumin induces arrest in the G_2_/M phase and mitotic catastrophe in three human cancer cell lines, while the reductive metabolism and chemical degradation inactivate the ability of curcumin to cause cancer cell death [18].

The relationship between curcumin and microbiota plays an important role in the gastrointestinal metabolism of curcumin [19]. The main metabolic process in human intestinal microbiota includes demethoxylation, reduction, hydroxylation, methylation, and acetylation [20]. A study used the bacteria strain, *Bacillus megaterium* DCMB-002, isolated from mice feces to treat with curcumin, and the bacteria have been found to transform curcumin into various metabolites [21]. In addition, the human intestinal bacteria, *Blautia* sp. MRG-PMF1, have been reported to biotransform curcumin via the methyl aryl ether cleavage reaction [22].

## 3. Bioactivity of Curcumin

### 3.1. Antioxidant Activity

The imbalance between free radicals and the body’s defense system against oxidative stress can cause various chronic diseases [23]. An excessive production of reactive oxygen species (ROS) can induce oxidative stress and damage essential biomolecules, while antioxidants, including antioxidant enzymes and antioxidant compounds, can protect the human body from free radicals and ROS effects, attenuating the progress of many chronic diseases [24]. Both *in vitro* and *in vivo* studies have revealed the antioxidant activity of curcumin contributes to its diverse therapeutic effects. The research on the chemical structure of curcumin shows that electron-donating groups of curcumin, especially the phenolic hydroxyl group, are the main contributors to its antioxidant activity [25].

Curcumin mainly reduces the oxidative stress by scavenging free radicals [26]. Studies have shown that curcumin can directly remove the excessive free radicals and prevent ROS production [27]. In A549 cells with influenza A virus (IAV)-induced oxidant stress, curcumin treatment is found to inhibit the production of ROS as well as the activation of toll-like receptor (TLR), which may be responsible for the suppression of influenza A virus infection [28]. Quinocetone causes genotoxicity and oxidative stress in human hepatocyte L02 cells, and curcumin pretreatment markedly inhibits excessive ROS generation, with the suppression of the decrease in the activity of antioxidant enzymes, like superoxide dismutase (SOD), and levels of antioxidant constituents, such as glutathione (GSH) [29]. In addition, in diabetic mice, curcumin treatment inhibits ROS production and hyperglycemia-induced oxidative stress by restoring the functions of DNA methyltransferase (DNMT) [30].

On the other hand, curcumin can also increase the activities of antioxidant enzymes. Treatment of curcumin significantly increases the activity of paraoxonase 1 arylesterase (PON1), reduces the susceptibility of low-density lipoprotein (LDL) oxidation, and restores the abnormal biochemical parameters caused by HgCl_2_ [31,32]. Additionally, the administration of curcumin results in amelioration of aflatoxin B1-induced effects via increases in the level of GSH, gene expression, and activities of antioxidant enzymes, such as catalase (CAT), SOD, glutathione peroxidase (GSH-Px), and glutathione-S-transferase (GST) [33]. Furthermore, curcumin significantly reverses the decreased activity of SOD induced by zymosan and attenuates the increase of the level of malondialdehyde (MDA) [34]. In allergic rhinitis, the curcumin fed rats show higher tissue GSH levels in inferior turbinate tissues and GSH-Px activity in serum than those of the control [35]. In addition, curcumin increases the GSH level in erythrocytes and plasma, while simultaneously decreases the oxidant potential of plasma [36]. Furthermore, an *in vitro* study indicates that curcumin blocks nuclear factor κB (NF-κB) activation due to its antioxidant activity [37].

Considering the potent antioxidant activity, curcumin has been found to scavenge free radicals, restore abnormal alternations induced by external factors, and repress transcription factors related to oxidation. These effects help reduce oxidative stress and lower the risk of various chronic diseases. However, it should be pointed out that a compound with antioxidant activity *in vitro* cannot represent an effective antioxidant *in vivo*, and it can be a pro-oxidant under certain conditions, showing no health benefits [38].

### 3.2. Anti-Inflammatory Activity

Chronic inflammation is caused by many external or intrinsic factors, and it is considered a key mediator for diseases. Curcumin can not only reduce the oxidative stress, but also protect against inflammation effectively via modulating pro-inflammatory cytokines and related signaling pathways, such as NF-κB, peroxisome proliferator-activated receptor-gamma (PPAR-γ), and myeloid differentiation protein 2-TLR 4 co-receptor (TLR4-MD2) signaling pathways [39,40,41,42].

#### 3.2.1. Regulation of Pro-Inflammatory and Anti-Inflammatory Cytokines

The inflammatory response is often accompanied by the excessive production of pro-inflammatory cytokines, such as interleukin-6 (IL-6), tumor necrosis factor-α (TNF-α), and interleukin-1β (IL-1β). Therefore, the downregulation of proinflammatory cytokines may effectively reduce the incidence of inflammation [43]. Pretreatment of curcumin on human genital epithelial cells abrogates the glycoprotein 120-mediated upregulation of the pro-inflammatory cytokines, TNF-α, and IL-6, as well as the chemokines, IL-8, RANTES (regulated on activation, normal T cell expressed, and secreted), and interferon γ-induced protein-10 (IP-10) [44]. Moreover, curcumin-loaded solid lipid nanoparticles can effectively decrease the expression of serum pro-inflammatory cytokines, including IL-6, TNF-α, and IL-1β [39]. Also, the liposomal curcumin complex effectively decreases pro-inflammatory cytokine and chemokine expression in synovial fibroblasts and macrophages without affecting cell viability, showing less toxicity compared to free curcumin [45]. The degradation product of curcumin, 4-vinyl guaiacol, has been reported to decrease IL-6 gene expression in lipopolysaccharide (LPS)-stimulated murine macrophages [46].

The upregulation of anti-inflammatory cytokines is also essential for the reduction of the inflammatory response. Results show that curcumin can inhibit inflammation and increase M2-like macrophages in white adipose tissues, promoting the production of anti-inflammatory cytokines [47]. Moreover, the *in vivo* study reveals that curcumin exhibits antiepileptogenic effects by upregulating the gene expression of anti-inflammatory cytokines, such as interleukin 10 receptor (IL-10R), chemokine (C-X-C motif) ligand 16 (CXCL16), and CXCL17 [48]. In cultured macrophages, it is observed that macrophages uptake the curcumin-loaded nanoparticles, and significantly increase the release of anti-inflammatory factors, including transforming growth factor-beta (TGF-β) and IL-10 [49]. To sum up, curcumin exhibits anti-inflammatory activity via the regulation of pro-inflammatory cytokines, such as IL-6, TNF-α, and IL-1β, as well as anti-inflammatory cytokines, such as IL-10 and TGF-β.

#### 3.2.2. Regulating Signaling Pathways Associated with Inflammation

Nuclear factor κB (NF-κB) is a critical inflammatory mediator that controls cytokine production and cell survival. Under the normal condition, NF-κB is in an inactive state by binding to an inhibitor of NF-κB (IκB) in the cytoplasm of most cells. Many inflammatory mediators, such as pro-inflammatory cytokines, chemokines, and leukocyte adhesion molecules, are upregulated during inflammation, and can activate NF-κB, which then translocates to the nucleus [50]. Curcumin has been found to exhibit anti-inflammatory activity by suppressing the NF-κB signaling pathway. A study shows that curcumin treatment maintains the *S*-nitrosylation of the inhibitor of NF-κB kinase subunit β (IKKβ, an activating kinase upstream of NF-κB) in dextran sulfate sodium-induced colitis. Sequentially, curcumin represses the phosphorylation of IκB and the activation of NF-κB [51]. In addition, pretreatment with curcumin shows an anti-inflammatory effect against the colistin-induced toxicity in neuroblastoma-2a cells, as it significantly downregulates the expression of the pro-inflammatory mediator cyclooxygenase-2 (COX-2), blocks the phosphorylation of IκB, and concomitantly decreases the NF-κB level [52]. Furthermore, the complex of polyethylene glycol (PEG) and curcumin inhibits NF-κB p65 nuclear translocation and c-Jun phosphorylation, and activation of nuclear factor (erythroid-derived 2)-like 2 (Nrf2), interfering with multiple targets involved in the inflammatory response [53]. Curcumin derivatives containing nonsteroidal anti-inflammatory components have been found to block the phosphorylation of IκB-α and suppress the activation of p65 and IκB-α [54]. The diarylpentadienone derivatives of curcumin inhibit LPS-induced inducible nitric oxide synthase (iNOS) expression, and slightly reduces the activation of p65 in nuclei [55]. Furthermore, curcumin analogs with different substitution groups decrease the expression of iNOS and COX-2, and inhibit NF-κB signaling in macrhahahophages [56].

**Figure 3:**
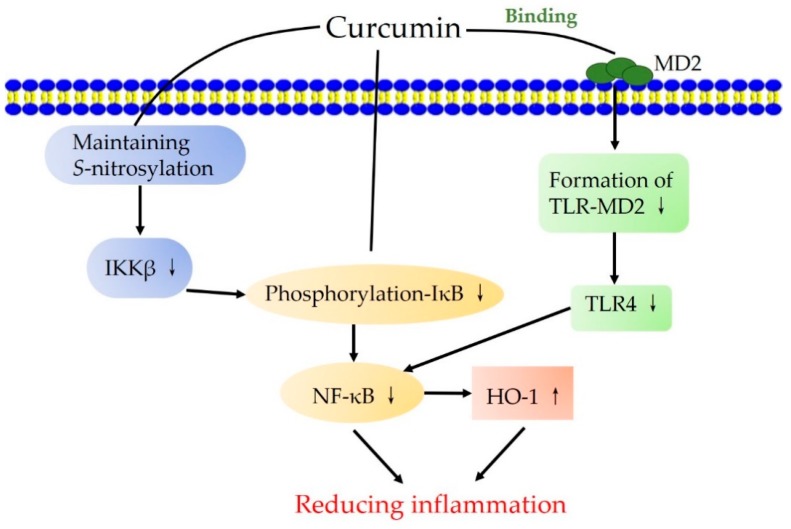
The signaling pathways involved in the anti-inflammation action of curcumin. The up arrows indicate the activation, while the down arrows indicate the inhibition. The treatment with curcumin induces the protection of S-nitrosylation on IKKβ, phosphorylation-IκB, and binding with MD2. The suppression of NF-κB activation and the increase of HO-1 activity contribute to the reduction of inflammation. HO-1, Heme oxygenase-1; IKKβ, Inhibitor of NF-κB kinase subunit β; IκB, Inhibitor of NF-κB; MD2, Myeloid differentiation protein 2; NF-κB, Nuclear factor κB; TLR4, Toll-like receptor 4; TLR4-MD2, Myeloid differentiation protein 2-TLR 4 co-receptor.

The anti-inflammatory property of curcumin is also involved in other signaling pathways. Curcumin is also found to induce degranulation in human neutrophils by increasing the cell surface expression of cluster of differentiation 35 (CD35) (secretory vesicle), CD63 (azurophilic granules), and CD66b (gelatinase granules) [40]. The control of neutrophils may be a potential anti-inflammatory mechanism of curcumin. In addition, curcumin exhibits anti-inflammatory activity via the PPAR-γ. A study finds that curcumin decreases the production of NO and suppresses the proliferation of vascular smooth muscle cells by elevating PPAR-γ activity, so as to attenuate angiotensin II-induced inflammatory responses [41]. Additionally, it has been reported that the TLR4-MD2 signaling complex is inhibited by curcumin and its analogs. It is supposed that curcumin and its analogs can compete with LPS for binding on MD2 and finally reduce the inflammation [42]. Overall, curcumin also exhibits anti-inflammatory activity by interacting with inflammation-related signaling pathways, such as NF-κB, PPAR-γ, and TLR4-MD2 signaling pathways (Figure 3).

### 3.3. Immune-Regulatory Activity

Numerous studies have indicated that curcumin is beneficial to the immune system, and it interacts with immune cells to protect against immune-related diseases by modulating various immune cells, such as various T lymphocyte subsets, macrophages, dendritic cells, B lymphocytes, and natural killer cells, and improving the aberrant alternations of immunological parameters [57].

Curcumin can reduce the numbers of neutrophil and eosinophil, and increase the lymphocyte counts [58]. In addition, Th1 cells produce IFN-γ, TNF-α, and IL-1β, while Th2 cells produce IL-4, IL-10, and TGF-β. Th17 cells can produce IL-17, and the immune suppressive Treg cells can prevent autoimmune disease. The balance of this subpopulation of T cells is crucial for the modulation of the immune system [59]. Meanwhile, pro-inflammatory M1 macrophages and anti-inflammatory M2 macrophages correspond to Th1 and Th2 cells, respectively. The levels of M1 and M2 macrophages are of great importance for the immune system homeostasis [60]. Curcumin poses a stimulatory effect on Th1 cells and an inhibitory effect on Th2 cells, regulating the Th1/Th2 balance in ovalbumin-sensitized rats. Curcumin exhibits protective effects against immunotoxicity induced by 2-amino-3-methylimidazole (4,5-f) quinoline, which diminishes the percentage of blood lymphocytes [61]. It has also been reported that curcumin attenuates myasthenia gravis via the regulation of various immune cells [62]. Specifically, it inhibits the expression of T cell co-stimulatory molecules, shifts the balance from Th1/Th17 toward Th2/Treg, increases the number of NKR-P1^+^ cells, and promotes the differentiation of B cells into a subset of B10 cells. Also, an *in vitro* study of bone marrow-derived mesenchymal stem cells with curcumin shows an increasing trend towards M2 macrophage polarization, providing a favorable immune microenvironment for cutaneous wound healing [60]. Moreover, curcumin mediates a lower level of macrophage infiltration and inhibits NF-κB activation in macrophages [63]. An *in silico* study indicates that the antioxidant property and strong binding affinity with CD4 and CD8 receptors of curcumin inhibit the thymic apoptosis induced by deltamethrin [64]. Furthermore, curcumin has been reported to provide a protection of immunity by reversing type-2 cytokine bias, reducing the Treg cell population, and inhibiting T cell apoptosis [65].

In fish fed with the dietary curcumin, it has been observed that the lysozyme activity, total IgG, and IgM levels increase after curcumin treatment, suggesting an effect on improving the immune responses to *Aeromonas hydrophila* [66]. For the weaned piglets orally administered with the enterotoxigenic *Escherichia coli*, the treatment of curcumin increases the secretory immunoglobulin (sIgA), improving the immune status [67]. Moreover, curcumin is able to modulate the production of immune mediators. For example, it can induce the increase of IFN-γ as well as the decrease of IL-1β, IL-4, IL-6, IL-17A, and TNF-α [58,67,68].

Overall, curcumin mainly improves the immune system via the interaction with immune cells, such as lymphocytes and macrophages. Also, curcumin induces immune responses by modulating immune molecules, such as IgG, IgM, and sIgA.

## 4. Health Benefits of Curcumin

### 4.1. Anticancer Effect

Over the past decade, cancer death rates have remained at a high level [69]. Curcumin is shown to have anticancer potential for the prevention and treatment of different cancers [70,71]. The underlying anticancer mechanisms of curcumin mainly depend on the inhibition of cancer cell growth, induction of cancer cell apoptosis, and suppression of cancer cell metastasis [70,72,73].

#### 4.1.1. Inhibition of Cancer Cell Growth and Proliferation

The observation on head-neck squamous cell carcinoma (HNSCC) cells indicates that the short-term exposure of curcumin at low concentrations results in the inhibition of colony formation as well as the growth of cells [73]. *In vitro* studies show that the treatment with curcumin causes 65% and 55% inhibition of cell proliferation in human nasopharyngeal carcinoma cell lines, CNE1 and CNE2, respectively [72]. In addition, curcumin treatment *in vivo* at the doses of 1000 and 1500 mg/kg leads to a 21.03% and 35.57% inhibition of the tumor growth, respectively, compared to the group of cervical cancer implanted nude mice [74]. Moreover, when curcumin is combined with other chemo drugs, like silymarin and paclitaxel, it shows a synergistic anticancer effect, exhibiting a higher inhibition on the growth of cancer cells [75,76].

The antiproliferative effect of curcumin is associated with the regulation of many signal pathways and transcription factors. Immunoblot analysis of HNSCC cells show that due to the exposure of curcumin, phosphorylation of NF-κB and signal transducer and activator for transcription 3 (STAT3) are reduced and the expressions of cyclin D1 and cyclin D2 are suppressed, contributing to the antiproliferative effect of curcumin [73]. In addition, it has been demonstrated that curcumin downregulates the expression of yes-associated protein 1 (YAP) and transcriptional coactivator with PDZ-binding motif (TAZ), two transcriptional co-activators with oncogenic effects, inhibits *Notch-1* gene expression, and reduces cell proliferation induced by the overexpression of YAP [77]. Furthermore, curcumin reduces cell proliferation through the regulation of miR-7/Skp2 by upregulating miR-7 expression and subsequently suppressing S-phase kinase-associated protein 2 (Skp2) [72]. In addition, curcumin pre-treatment has been observed to inhibit the growth of breast cancer cells in rats via activation of PPAR-γ and decreasing the expression of brain-derived neurotrophic factor (BDNF) [78]. Moreover, an *in vivo* study shows that curcumin administration results in the downregulation of proliferating cell nuclear antigen (PCNA) as well as STAT3 expression [79]. Additionally, it is observed that nano-curcumin is effective in suppressing the proliferation of esophageal squamous cell carcinoma KYSE-30 and downregulating the expression of cyclin D1 [80].

Interference of the cell cycle inhibits cellular proliferation in various cancers. Curcumin induces the cell cycle arrest accompanied with irregular alterations in the number of cells in different phases, such as the G_1_/S and G_2_/M phases [81,82]. In Ras-activated HAG-1 human adenocarcinoma cells, curcumin treatment increases the proportion of cells in the G_2_/M phase from 20% to 52%, and decreases the proportion of cells in the S and G_0_–G_1_ phases, suggesting that the arrest in the G_2_/M phase can mediate a growth decline [83]. In Patu8988 pancreatic cancer cells, the accumulation of cell population in the G_2_/M phase increases from 15.07% to 19.59% and 23.75% with 10 and 15 μM of curcumin, respectively [77]. The cell cycle analysis shows that the G_2_/M phase fraction is increased from 7.29% to 38.99% and 51.94% with 5 µM and 10 µM of curcumin in the CNE1 cells, respectively [72]. Moreover, the synergistic effect of curcumin and diclofenac has been found to inhibit telomerase activity and upregulate the tumor suppressor proteins, p51, Rb, and p21, in colorectal cancer with the induction of cell cycle arrest [84]. In addition, apigenin and curcumin synergistically block the cell cycle progression in the G_2_/M phase of A549 cells with an increase of the expression of p53 [85].

Angiogenesis is strictly controlled in normal tissues, while it loses control in the tumor and promotes tumor formation. Curcumin is considered as an angiogenesis inhibitor, and leads to tumor regression and inhibition of tumor growth [86]. A high dose of curcumin reduces cervical cancer-induced increase in capillary networks and attenuates abnormalities of the capillary network pattern [74]. These effects are probably related to the downregulation of angiogenic biomarkers, including vascular endothelial growth factor (VEGF), COX-2, and epidermal growth factor receptor (EGFR). Additionally, the combination of doxorubicin and curcumin in pH-sensitive nanoparticles constituted with amphiphilic poly (β-amino ester) copolymer exert anti-angiogenesis effect by increasing the expression phosphor-VEGF receptor 2 and the suppression of all signaling proteins related to VEGF-induced pathways [87].

#### 4.1.2. Induction of Cancer Cell Apoptosis

Curcumin has exhibited anticancer effects via inducing apoptosis in a variety of cancers. The apoptosis induced by curcumin leads to the morphological alternations of cancer cells, including cell shrinkage, cytoplasmic blebbing, irregularity in shape, and externalization of cell membrane phosphatidylserine [88]. The apoptosis mechanism of curcumin involves certain biomolecules and several signal pathways [89]. The derivates and complexes of curcumin can also induce the apoptosis of cancer cells, which may provide more application possibilities in cancer therapy [90].

Bcl-2 family proteins are key mediators in the regulation of cell death by inhibiting or inducing apoptosis. The anti-apoptotic proteins mainly contain B-cell lymphoma-2 (Bcl-2), Bcl-xL, and myeloid cell leukemia 1 (Mcl-1), while Bcl-2-associated X protein (Bax), Bcl-2 homologous antagonist killer (Bak), Bcl-xS, and Bcl-2-interacting killer (Bik) are pro-apoptotic proteins. In Src-activated HAG-1 human adenocarcinoma cells, curcumin inhibits cell growth by suppressing the level of Bcl-xL and activating the expression of Bax [83]. Curcumin promotes apoptosis in HNSCC cells by upregulating pro-apoptotic Bik prior to the suppression of cell proliferation [73]. Moreover, curcumin has been demonstrated to downregulate *Mcl-1* gene expression in MCF-7 breast cancer cells, decreasing the viability of cancer cells [91]. Additionally, the combination of paclitaxel and curcumin increases anti-tumor efficacy in mouse models, reduces the expression of Bcl-2, and increases the expression of Bax [75].

Regulation of apoptosis in cancer cells is associated with modulation of several signaling pathways. Curcumin has been reported to decrease the viability of human lung cancer A549 cells and induce apoptosis as well as autophagy [70]. Apoptosis and autophagy by curcumin are related to the downregulation of phosphatidylinositol 3-kinase (PI3K)/protein kinase B (Akt)/mammalian target of rapamycin (mTOR) pathway. In ovarian cancers, there is an abnormal increase in the expression of sarco/endoplasmic reticulum calcium ATPase (SERCA) [92], which can regulate Ca^2+^ homeostasis, and curcumin treatment has been found to suppress SERCA activity, leading to the disruption of Ca^2+^ homeostasis, and apoptosis of ovarian cancer cells. Recent studies demonstrate that the activity of leucine-zipper and sterile-alpha motif kinase alpha (ZAKα) is inhibited by curcumin, suggesting the involvement of apoptosis induction by curcumin [93]. Also, in colorectal cancer cells, curcumin increases p53 expression in tumor cells and modulates the tumor cell apoptotic pathway, promoting apoptosis of tumor cells [94]. Several studies indicate that curcumin and its analog can suppress the expression of miR-21, cyclin D1, and antigen Ki-67, but enhance the expression of phosphatase and tensin homolog (PTEN), programmed cell death protein 4 (PDCD4), and miR-21 target genes, resulting in induced apoptosis and antiproliferation in cancer cells [95,96]. Additionally, the polymeric nano-encapsulation of curcumin reduces the expressions of hypoxia-inducible factor 1-α (HIF 1-α) and nuclear p65 (Rel A), and subsequently induces apoptosis in breast and lung cancer cells [97].

The combination of curcumin and other components exhibits synergetic anticancer activity on inducing apoptosis of various cancer cells. On cell line models (A549, Hep-G2, MCF-7, Jurkat, and K562), the combination of berberine and curcumin causes more than 77% cell death, while the curcumin alone or berberine alone causes less than 54% or 45% cell death, respectively [98]. Compared with only curcumin or silymarin treatment, the combination treatment of curcumin and silymarin induces five-fold higher caspase 3/7 activities and a higher level of apoptosis in colon cancer cells [76]. In addition, the co-treatment of curcumin with cisplatin triggers ROS production, caspase-3 activation, and upregulation of p-ERK1/2 (ERK, Extracellular signal-regulated kinases), increasing the apoptosis rate of bladder cancer cells [99].

Curcumin is also considered as an auxiliary agent, and intensifies the therapeutic effects of other cancer treatments. When used in irradiation therapy, curcumin prevents radiation-induced activation of the NF-κB pathway, increases sensitivity to ionizing radiation and apoptosis of tumor cells, and decreases tumor cell proliferation, improving anticancer efficacy [100]. Moreover, the addition of curcumin is found to potentiate 5-fluorouracil-induced reduction of proliferation and invasion in colorectal cancer cells [101], and curcumin enhances the chemosensitivity of colorectal cancer cells to 5-fluorouracil and induces apoptosis. These effects are speculated to be associated with the downregulation of NF-κB activation, inhibition of AMP-activated protein kinase (AMPK)/unc-51-like kinase (ULK1)-dependent autophagy, and the suppression of Akt activity [102].

#### 4.1.3. Suppression of Cancer Cell Metastasis and Invasion

Metastasis and invasion promote the spread and growth of various cancer cells. Several studies have reported that curcumin exhibits its anticancer activity by suppressing metastasis and invasion progression of cancer cell lines, which can be a promising therapeutic target for cancers [103]. Anti-metastatic and anti-invasive mechanisms of curcumin are involved in the inhibition of transcription factors, inflammatory cytokines, proteases, protein kinases, and the regulation of miRNAs [104]. Wound healing and transwell assays show that following curcumin treatment, metastasis and invasion into the Matrigel-coated membrane of nasopharyngeal carcinoma (NPC) cells are remarkably inhibited [72]. Compared with pure curcumin, catanionic lipid nanosystems incorporating curcumin show a 1.8-fold higher anti-invasion capacity on Lewis lung cancer cells [105]. Polymeric micelles of curcumin prepared in a thermosensitive hydrogel system have been identified to possess higher inhibitory effects against tumors in the colorectal peritoneal carcinomatosis mouse model, and the growth and metastasis of tumors are suppressed by the curcumin complexes, and the survival of tumor-bearing mice is prolonged [106].

**Figure 4:**
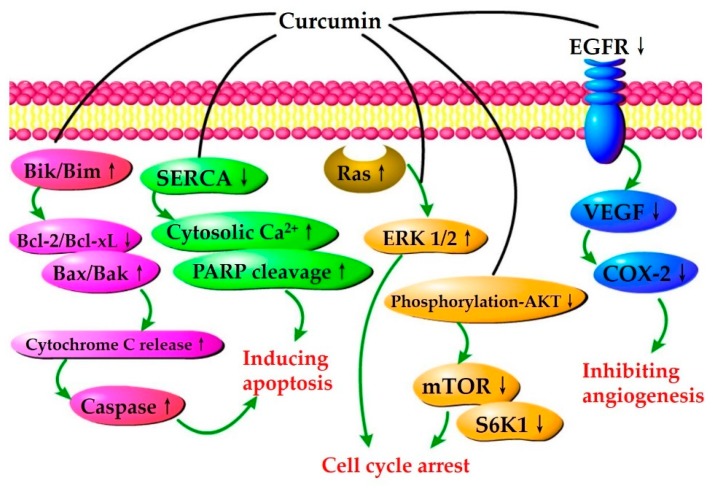
Curcumin-mediated anticancer signaling pathways, associated with the induction of apoptosis and cell cycle arrest, as well as the inhibition of angiogenesis. The up arrows indicate the upregulation, while the down arrows indicate the downregulation. Bcl-2, B-cell lymphoma 2; Bcl-xL, B-cell lymphoma-extra large; Bik, Bcl-2-interacting killer; Bim, Bcl-2 interacting mediator of cell death; Bax, Bcl-2-associated X protein; Bak, Bcl-2 homologous antagonist killer; SERCA, Sarco/endoplasmic reticulum calcium ATPase; PARP, Poly (ADP-ribose) polymerase; Ras, an oncogene product in many human cancers; ERK1/2, Extracellular signal-regulated kinases; Akt, Protein kinase B; mTOR, Mammalian target of rapamycin; S6K1, p70-S6 kinase 1; EGFR, Epidermal growth factor receptor; VEGF, Vascular endothelial growth factor; COX-2, Cyclooxygenase-2.

Collectively, curcumin has shown anticancer effects on various cancer cells, such as breast, lung, and colon cancer cells [107]. As shown in Table 1 and Figure 4, the mechanisms of action are mainly associated with cell growth, proliferation, angiogenesis, apoptosis, metastasis, and invasion [108]. In addition, the association of curcumin with other components and anticancer therapy has a synergic effect to improve the prevention and treatment of cancers.

### 4.2. Hepatoprotection

Numerous studies indicate that curcumin exhibits hepatoprotective effects on acute and chronic liver injuries induced by pollutants, drugs, and alcohol, nonalcoholic fatty liver disease, and liver fibrosis [109,110].

#### 4.2.1. Liver Injuries Induced by Pollutants, Drugs, and Alcohol

Carbon tetrachloride (CCl_4_), a well-known hepatotoxin pollutant, can induce acute or chronic liver injuries, and result in a high level of ROS, mitochondrial dysfunction, and cellular apoptosis [111]. Nanoparticulated curcumin has been evidenced to maintain cellular ROS levels, increase cellular antioxidant enzymes, prevent excessive mitochondrial destruction, and decrease fatty changes and inflammation in CCl_4_-treated rat hepatocytes [112]. Enzymes, such as aspartate aminotransferase (AST) and alanine transaminase (ALT), in serum, are the main liver transaminases for liver damage assessment, and the hepatic GSH content indicates oxidative stress in the liver [113]. Turmeric extract and curcumin are found to suppress CCl_4_-induced hepatic oxidative stress. They decrease the activities of serum aspartate aminotransferase (AST) and alanine transaminase (ALT) and the level of lipid peroxidase, while increasing the hepatic GSH content [114]. Additionally, curcumin is able to reduce the number of apoptotic hepatocytes induced by cadmium accumulation [115].

The intake of overdose drugs may cause severe side-effects, like hepatoxicity. Curcumin has been reported to ameliorate streptozotocin-induced liver injury of diabetic rats by decreasing hepatic endoplasmic reticulum stress marker protein and the sub-arm of unfolded protein response signaling protein, and inhibiting TNF-α, IL1β, phospho-p38 MAPK, and ASK1 in liver [116]. Paracetamol-induced mitochondrial dysfunction in hepatocytes is also attenuated by curcumin. It decreases oxygen consumption in the membrane potential, ATP synthesis, aconitase activity, and the activity of respiratory complexes, I, III, and IV [117].

Alcoholic liver disease is characterized by the disturbance of alcohol and lipid metabolism. A metabolomic pathway analysis shows that curcumin attenuates ethanol-induced liver injury by inhibiting the biosynthesis of unsaturated fatty acids, and interconversions of pentose and glucuronate [118]. In addition, curcumin dose-dependently attenuates the hepatocyte necroptosis and alcohol-induced decrease in hepatic Nrf2 expression in alcoholic mice [119].

#### 4.2.2. Nonalcoholic Fatty Liver Disease

Nonalcoholic fatty liver disease (NAFLD) is acknowledged as a hepatic disease with multiple risk factors [120]. NAFLD is often accompanied by changes in metabolic and non-metabolic processes, like dyslipidemia, inflammation, and oxidative stress. Recently, curcumin has been reported to possess therapeutic efficacy in NAFLD experimental models, including non-alcoholic steatohepatitis (NASH) [121]. Oral administration of curcumin effectively protects against the progression of NAFLD induced by a high-fat diet through alternations in the metabolism, and the intrahepatic CD4^+^ cell accumulation [122]. Furthermore, high mobility group box 1 (HMGB1) is demonstrated to interact with TLR4, and induce nuclear translocation of activated NF-κB, releasing pro-inflammatory cytokines [123]. Curcumin can reduce cytoplasmic translocation of HMGB1, the level of TLR4 protein expression, and the nuclear translocation of NF-κB [124]. Moreover, some studies elucidate that curcumin exhibits its hepatoprotective property by inhibiting transient receptor potential melastatin 2 (TRPM2) channels, and curcumin can restore Ca^2+^ levels, reduce oxidative stress, and lower the risk of NASH [125].

#### 4.2.3. Liver Fibrosis

Liver fibrosis is the excessive accumulation of scar tissue induced by inflammation and liver cell death in chronic liver diseases, and it is affected by various factors, like overconsumption of alcohol or exposure to toxic chemicals. The hepatic stellate cell (HSC), the major fibrogenic cell in the liver, is involved in fibrosis, forming scar tissue in response to liver damage. Administration of curcumin has been reported to reduce the viability of HSCs by inhibiting the proliferation and stimulating endoplasmic reticulum stress [126]. Curcumin also suppresses the TGF-β/Smad signaling pathway and blocks the production of extracellular matrix proteins in HSCs. A novel target for liver fibrosis treatment is to modulate the cannabinoid receptors (CBRs) system. Curcumin can also reduce the extracellular matrix overproduction in HSCs through the downregulation of CBR type 1 and upregulation of CBR type 2 [127]. Further investigations reveal that curcumin inhibits the angiogenesis in liver fibrosis. Curcumin reduces the angiogenic properties of HSCs, disrupts the platelet-derived growth factor- receptor (PDGF-R)/ERK and mammalian target of rapamycin (mTOR) pathways, and regulates activation of PPAR-γ [128]. In addition, curcumin can suppress liver fibrosis via miRNA-mediated epigenetic regulation. It has been reported that curcumin upregulates miR-29b expression with the downregulation of DNA methyltransferase 3b as well as the upregulation of PTEN, which inhibits the activated HSCs [129]. Furthermore, details of the hepatoprotective activity of curcumin are also shown in Table 2.

### 4.3. Neuroprotection

Curcumin has shown the potential as a neuroprotective agent due to its antioxidant and anti-inflammatory activities and the ability to maintain the chemical balance in the brain [26]. Certain investigations reveal that curcumin pretreatment is effective to reduce H_2_O_2_-induced neurotoxicity in PC12 cells by attenuating caspase activation, poly (ADP-ribose) polymerase (PARP) cleavage, DNA damage, and the accumulation of ROS, and dysregulation of the MAPK and Akt pathways. These protective effects can be used in the therapy for human neurodegenerative diseases [130]. Furthermore, the neuroprotective property of curcumin can reduce H_2_O_2_-induced damage in the SH-SY5Y human neuroblastoma cell line, and enhance cell viability [131]. Anti-neuroinflammatory results elucidate that curcumin inhibits the secretion of pro-inflammatory mediators and cytokines, induces HO-1 transcription and translation, and modulates NF-κB and MAPK signaling pathways, reducing inflammation in microglial cells [132]. The suppression of inflammation by curcumin leads to the reduction in astrocyte hypertrophy and the activation of microglia in the hippocampus, contributing to a better memory and mood function [133]. Furthermore, the pretreatment of curcumin is also effective to attenuate brain trauma and improve neurological function, alleviate neuropathic pain, mitigate axonal injury, and protect against ischemia and reperfusion injury [134,135,136,137,138].

*In vitro* and *in vivo* studies have shown the potential of curcumin as an adjuvant for the treatment of neurodegenerative diseases, such as Alzheimer’s disease (AD) and Parkinson’s disease (PD). In neurodegenerative diseases, curcumin treatment can bind to some proteins or limit the aggregation of other protein to maintain homeostasis of the inflammatory system and facilitate the heat shock system [139]. Numerous studies acknowledge that the accumulation of amyloid-β protein and hyperphosphorylated tau proteins is the hallmark of AD. Curcumin may directly bind to PPAR-γ and increase transcriptional activity and protein levels of PPAR-γ, which alleviates amyloid-β-induced neuroinflammation and improves neuronal status in the rat model of AD [140]. The toxicity of the amyloid-β peptide damages spatial learning and memory through the c-Jun N-terminal kinase (JNK) signaling pathway in AD. After treating with curcumin, AD rats show restored spatial learning and memory deficits, and JNK-3 phosphorylation is inhibited [141]. In mice with AD, active avoidance and locomotor activity are improved by curcumin, which protects against neurodegeneration [142]. For PD, increasing evidence indicates that the accumulation of α-synuclein protein plays a vital role in the occurrence of PD [143], suggesting that curcumin can reduce the accumulation of A53T α-synuclein by downregulating the mTOR/ribosomal protein S6 kinase (p70S6K) signaling, and restoring macroautophagy. Curcumin prevents α-synuclein from aggregating in the dopaminergic neurons at both the gene level and protein expression level [144]. Meanwhile, curcumin inhibits astrocytic activation, and reduces iron deposition on the dopaminergic neurons. Moreover, the cognitive function is impaired in mice with PD, while curcumin treatment in mice reduces the enhanced acetylcholine esterase enzyme levels, and restores the moto deficits, improving the cognitive functions [145]. Curcumin provides protection for neurons by reducing p-p28 expression, caspase-3 activation, and toxic quinoprotein formation in 6-hydroxydopamine treated SH-SY5Y cells, ameliorating oxidative stress-related neurodegeneration, like PD [146].

It has been documented that the exposure to some heavy metals, medications, and chemicals can induce nerve injuries, and even neuropathology of several neurodegenerative disorders. Curcumin diminishes copper-induced neurotoxicity *in vivo* by upregulating tyrosine hydroxylase expression and restoring locomotor performance [147]. In addition, curcumin treatment can ameliorate arsenic-induced mitochondrial dysfunctions and modulate proteins related to apoptosis to reduce cholinergic deficits [148]. Additionally, curcumin has been reported to abrogate the neurotoxicity induced by some drugs. For example, curcumin is found to remarkably ameliorate the abnormal alternations caused by cisplatin, including thermal hypoalgesia, reduced sciatic motor nerve conduction velocity, and nuclear and nucleolar atrophy with the loss of neurons [149]. Bupivacaine, a widely-used anesthetic, has neurotoxicity in SH-SY5Y cells, and curcumin has been observed to protect against neuronal injury by increasing the level of Akt phosphorylation [150]. With the intervention of curcumin on pregnant mice, cultured neurons, and neural progenitor cells, results show that curcumin can mitigate the toxic effects of celecoxib on fetal brain development by suppressing the proliferation of neuronal progenitor cells via activation of the Wnt/β-catenin pathway [151]. Furthermore, the treatment of curcumin has been reported to reduce colistin-induced neurotoxicity in neuroblastoma-2a through regulating NF-κB signaling, leading to antioxidative and anti-apoptotic responses [52]. Furthermore, toxic chemicals, like quinolinic acid, also impair the nervous system. Curcumin prevents rotation behavior, striatal morphological alterations, and neurodegeneration induced by quinolinic acid, and activates the Nrf2 cytoprotective pathway to induce antioxidant responses [152].

### 4.4. Cardiovascular Protection

Cardiovascular diseases (CVDs) have threatened human health severely worldwide. It has been documented that the intake of curcumin is beneficial to CVDs [153,154].

Curcumin can protect cardiomyocytes as a cardio-protectant. For instance, curcumin protects against norepinephrine-induced hypertrophic stress and maintains extracellular matrix remodeling by inhibiting the increased collagen content under hypertrophic stress, and suppressing enhanced gelatinase B expression and gelatinolytic activity in H9c2 cardiomyocytes [155]. It has also been found that curcumin prevents cardiomyocytes from norepinephrine-induced apoptosis and restores their physiological status [156]. In addition, the administration of curcumin can attenuate the cytotoxicity of hemiscorpius lepturus venom, which damages the mitochondrial respiratory chain and results in ATP depletion, death signaling, and consequent cardiomyocytes apoptosis [157]. Furthermore, curcumin increases the viability of H9c2 cardiomyoblasts exposed to H_2_O_2_ by upregulating the HO-1 protein, inhibiting cleaved caspase-3 protein expression, and increasing the Bcl-2/Bax ratio [158]. Additionally, curcumin prevents cardiomyocytes from high glucose-induced apoptosis via the inhibition of NADPH-mediated oxidative stress by modulating the PI3K/Akt signaling pathway [154].

It is reported that curcumin can ameliorate cardiac hypertrophy and preserve cardiac function by upregulating Na^+^/Ca^2+^ exchanger expression levels. The left ventricle (LV) end-systolic and diastolic dimensions are reduced, and the LV ejection fraction and LV fractional shortening are enhanced *in vivo* by curcumin [159]. Another work uses high doses of glucose and insulin to induce hypertrophy in cardiomyocytes and the treatment of curcumin decreases the increased ANF mRNA expression, total protein content, and cell surface area, resulting in attenuated cardiomyocyte hypertrophy [160]. Meanwhile, curcumin increases PPAR-γ and Akt protein expressions, endothelial NO synthase (eNOS) mRNA expression, eNOS content, and NO concentration in cardiomyocytes. Curcumin is also found to suppress hypertrophic responses in cardiomyocytes by disrupting the formations of the zinc finger transcription factor, GATA4, and functional proteins, such as intrinsic histone acetyltransferase, and p300 [161]. Additionally, curcumin can attenuate chronic heart failure by increasing Dickkopf-related protein 3, p38 MAPK, JNK, and apoptosis signal-regulating kinase 1 (ASK1) [162]. Moreover, curcumin has been demonstrated to have hypocholesterolemic activity both *in vitro* and *in vivo*. Curcumin treatment is found to reduce cholesterol absorption in Caco-2 cells via downregulation of expressions of Niemann-Pick C1-like 1 (NPC1L1) and sterol regulatory element binding protein-2 (SREBP-2) [163].

The occurrence of CVDs is closely related with unbalanced diets. For curcumin, it protects the cardiomyocytes from injuries, and decreases cardiac hypertrophy, chronic heart failure, and cholesterol absorption.

### 4.5. Anti-Diabetic Effect

Diabetes mellitus (DM) has reached pandemic status and there have been numerous studies on the development of anti-diabetic drugs with fewer side effects [164]. Curcumin has been found to exhibit promising anti-diabetic activity, and curcumin intervention on prediabetic individuals can significantly lower the risk of developing type 2 DM [165]. Additionally, curcumin can be a potential alternative to prevent and treat diabetes as well as certain complications, such as diabetic retinopathy [166,167].

The anti-diabetic activity of curcumin commonly reflects on the control of hyperglycemia by downregulating α-glucosidase and α-amylase activity. In addition, curcumin is beneficial for insulin-producing and insulin-responsive tissue, such as liver, skeletal muscle, and adipose tissues [168]. Molecular docking *in silico* shows that curcumin poses a better inhibitory effect on α-amylase than other natural compounds, such as quercetin and berberine [169]. After hyperglycaemic rats were administered with curcumin, the mean blood glucose level was significantly reduced [170]. Additionally, the glucose tolerance and insulin sensitivity of diabetic rats were enhanced after being treated with curcumin [171]. Meanwhile, the levels of Akt phosphorylation and glucose transporter type 4 (GLUT4) translocation in skeletal muscles are increased by curcumin. Moreover, in diabetic mice treated with curcumin for 12 weeks, pancreatic islets have no lymphocytes infiltration and there is an increase in the numbers of small islets of Langerhans near the ducts in the pancreas, which indicates an improvement of pancreatic islets [172].

### 4.6. Other Health Benefits

**Figure 5:**
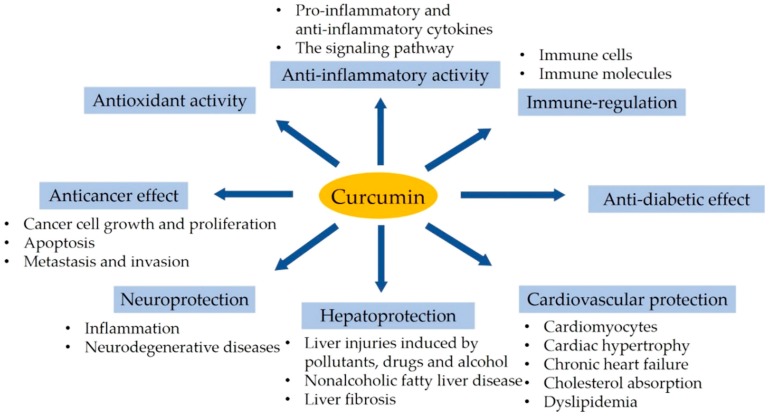
A summary of the bioactivity and health benefits of curcumin, including antioxidant, anti-inflammatory, immune-regulatory, anticancer, neuroprotective, hepatoprotective, cardiovascular protective, and anti-diabetic effects.

Curcumin has been documented to pose preventive and therapeutic effects on other diseases. For instance, curcumin exhibits anti-influenza virus activity, and it can inhibit type A influenza virus (IAV) infection through interference with the receptor binding region of viral haemagglutination (HA) protein, severely affecting viral HA activity [173]. Moreover, the results of blood compatibility tests demonstrate the antiplatelet property of curcumin. Fewer platelets adhered and aggregated on the surface with an increase in the content of curcumin [174]. Furthermore, chronic exposure to curcumin has been found to alleviate seizure activity induced by pentylenetetrazole in rats. It has also been observed that chronic curcumin pre-treatment increases the myoclonic jerks latency, while decreasing the duration of generalized tonic-clonic seizures and the severity of seizure [175]. Finally, the bioactivity and health benefits of curcumin are summarized in Figure 5.

## 5. Clinical Trials

In the past five years, some clinical trials have been conducted to investigate the effects of curcumin on patients with several diseases, such as metabolic syndromes, diabetes, arthritis, and gut inflammation [176]. In clinical trials, curcumin is often used in combination with other agents or alone with delivery systems, such as nanoparticles and liposomal encapsulation [177,178].

Oxidative stress plays an important role in the progression of nondiabetic or diabetic proteinuric kidney disease. Specifically, a randomized double-blind placebo-controlled clinical trial was performed to evaluate the antioxidant effects of curcumin on chronic kidney disease (CKD). One hundred and one patients with nondiabetic or diabetic proteinuric CKD received a placebo or 320 mg of curcumin per day for eight weeks. Results showed that curcumin attenuates lipid peroxidation in plasma in patients with nondiabetic proteinuric CKD and improved the antioxidant capacity in patients with diabetic proteinuric CKD [179].

Due to its anti-inflammatory activity, curcumin could provide an improvement on inflammatory diseases. Thirty-six patients with rheumatoid arthritis (RA, a chronic systemic inflammatory disorder) were randomly assigned to receive a placebo, 250mg, or 500 mg of curcumin twice daily for 90 days. At the end of the study, the clinical markers of inflammation, including erythrocyte sedimentation rate (ESR) and C-reactive protein (CRP) values, were significantly improved in the groups receiving curcumin [180]. In addition, the effects of curcumin on osteoarthritis (OA) were evaluated, and 22 patients with knee OA took three caps of bio-optimized curcumin twice a day. The treatment with curcumin significantly reduced the serum levels of Coll2-1, a cartilage-specific biomarker, attenuating knee OA [181]. Psoriasis vulgaris is a common chronic inflammatory disease. Sixty-three patients with mild-to-moderate psoriasis vulgaris were treated with 2 g of topical steroids and oral curcumin per day, or with topical steroids alone for 12 weeks. In patients treated with oral curcumin, the psoriasis area and severity index (PASI) values and serum levels of IL-22 were significantly reduced and the inflammation progression was ameliorated [182]. Similarly, patients with moderate-to-severe psoriasis were treated orally with 0.4 mg/kg of acitretin and 3 g of nanocurcumin or only with acitretin every day for 12 weeks. It resulted in reduced PASI values and unchanged cholesterol serum levels in patients treated with curcumin and acitretin [177]. Furthermore, migraines are characterized by high levels of IL-6 and CRP, which disrupt the integrity of the blood-brain barrier and induce neurogenic inflammation. Eighty episodic migraine patients were randomized to receive a combination of ω-3 fatty acids (2500 mg) and nanocurcumin (80 mg) or ω-3 alone or nanocurcumin alone for two months. Notably, the combination of nanocurcumin and ω-3 downregulated IL-6 mRNA and decreased the serum levels of IL-6 as well as high sensitivity CRP (hs-CRP) [183].

Curcumin supplements are considered as a preventive agent and an adjuvant therapy of cancers. Oral leukoplakia is a potentially malignant lesion of the oral cavity. Subjects with oral leukoplakia (*n* = 223) randomly took orally 3.6 g of curcumin (*n* = 111) or a placebo (*n* = 112) daily for six months. The primary endpoint was a clinical response obtained by bi-dimensional measurement of the leukoplakia size at six months of recruitment. At the end of the study, the treatment with curcumin had no safety concerns and the subjects were well tolerated and given a durable clinical response for six months [184]. Additionally, curcumin has been reported to have antitumor effects on glioblastoma cells in human. Thirteen glioblastoma patients received 70 mg of micellar curcuminoids, consisting of curcumin, demethoxycurcumin, and bis-demethoxycurcumin, three times per day for four days. After the intervention of curcuminoids, the mean ratio of phosphocreatine to inorganic phosphate decreased, and the mean intratumoral pH increased, attenuating intratumoral energy metabolism [185].

In clinical trials, curcumin has also been demonstrated to show protection against cardiovascular diseases. The progression of atherosclerosis can be accelerated by alpha 1-antitrypsin-low-density lipoprotein (AT-LDL). Subjects with stages I-II chronic obstructive pulmonary disease (COPD) were randomly treated with 90 mg of Theracurmin (R) (a highly absorptive curcumin using a drug delivery system) or a placebo twice a day for 24 weeks. The level of AT-LDL was significantly lower in the curcumin group compared with the placebo group [186]. The curcumin extract was evidenced to have a lipid-lowering effect via a clinical trial of 65 patients with metabolic syndromes. Patients were randomized into two groups to take a 630 mg curcumin extract capsule or a placebo capsule thrice daily for 12 weeks. The level of high-density lipoprotein cholesterol (HDL-C) were significantly increased, and the levels of LDL and triglycerides were significantly reduced after curcumin extract treatment for 12 weeks [187]. A six-month clinical trial with type 2 diabetic patients shows that curcumin intervention significantly reduces pulse wave velocity, increases the level of serum adiponectin, and decreases the level of leptin, lowering the atherogenic risks [188]. A randomized double-blind placebo-controlled trial including 33 patients with coronary artery disease revealed that the intake of curcumin significantly ameliorates dyslipidemia in patients. Curcumin was observed to control the blood lipid levels by decreasing the serum levels of triglycerides, LDL-cholesterol, and very low density lipoprotein-cholesterol (VLDL-C) [189].

Curcumin supplementation may exert beneficial effects on the management of diabetes. In an open-label, randomized control trial, eight type-2 diabetic patients were treated with curcumin capsules on glyburide therapy for 10 days. Results show that the levels of blood glucose, LDL, VLDL, and triglycerides were decreased significantly, and the content of HLDL was increased. The findings indicate that the combination of curcumin capsules with glyburide contributes to better glycemic control in diabetic patients [190]. In addition, 70 type-2 diabetic patients randomly received 80 mg of nano-micelle curcumin or a placebo daily for three months, and in the group with nano-micelle curcumin, a significant decrease was observed in glycated hemoglobin (HbA1C) and fasting blood glucose (FBG) which are biochemical parameters related to diabetes [191].

Overall, several clinical trials have demonstrated the beneficial effects of curcumin in patients with inflammation, cardiovascular diseases, metabolic syndrome, or diabetes.

## 6. Challenges and Perspectives

Curcumin has been documented to exhibit therapeutic or protective effects against a wide spectrum of diseases, but curcumin is unstable under various conditions and is easily degraded or metabolized into other forms. For instance, the dose of curcumin given orally is 8 g/day in humans, and the data shows that curcumin is rapidly transformed into metabolites, resulting in a low level of free curcumin in plasma (<2.5 ng/mL) [192]. The poor aqueous solubility and low bioavailability of curcumin are considered as the main obstacles to its clinical development, and the metabolites and derivatives may be responsible for the biological activities rather than free curcumin [7]. To enhance the stability and bioavailability of curcumin, many studies focus on modifications of curcumin and its delivery systems, such as nanoparticles, micellation, and conjugation with other materials [193,194,195]. Such modifications can increase its stability, solubility, *in vivo* uptake, bioactivity, and safety [196,197,198,199,200].

Recently, there have been different points of view on the beneficial effects of curcumin. It is argued that the therapeutic benefits of curcuminoids are skeptical, since they are unstable and nonbioavailable [2]. In addition, it is claimed that curcuminoids may interact with proteins to give false signals in drug screening tests, and the focus on curcuminoids is a waste of sources [201]. However, it is also considered that scientists should logically study natural products rather than asserting their inefficacy one-sidedly [202], as curcumin might be an important adjunctive treatment for certain diseases. In addition, it is stated that the clinical trials of curcumin should not be dismissed, curcumin really exhibits therapeutic benefits, and the binding of curcumin with other molecules is modulation rather than inhibition [203]. It is also emphasised that more large trials with controlled placebos are needed to verify the therapeutic effects of curcumin on the human body [204]. In light of the instability of curcumin, although its efficacy has been challenged, many animals and human studies, especially preclinical trails, still support the therapeutic and protective effects of curcumin, while more clinical trials are necessary to clarify its effects on human.

## 7. Conclusions

In conclusion, curcumin has been reported to exhibit versatile bioactivity and provide diverse health benefits to humans, such as antioxidant, anti-inflammatory, and anticancer activities. In addition, there are increasing studies on finding novel molecular targets of curcumin, and its mechanisms of actions are expected to be understood more clearly. Furthermore, it is promising to use the nanotechnology to encapsulate curcumin to improve its stability, bioavailability, bioactivity, and health benefits. Although there is still a debate about its beneficial effects on humans, numerous preclinical studies still support its health benefits due to their versatile bioactivity. Therefore, curcumin can be still a good natural ingredient for the development of relevant functional foods as promising alternatives for the prevention of certain chronic diseases.

## Figures and Tables

**Table 1:** Anticancer activity and related molecular mechanisms of curcumin.

Study Type	Subjects	Dose	Potential Mechanisms	Ref.
***Effects on cancer cell growth and proliferation***				
*In vitro*	HNSCC cells	12.5 μM	Upregulating pro-apoptotic Bik Downregulating survival signaling by Akt and NF-κB Reducing STAT3 expression Suppressing cyclin D1 and cyclin D2 expression	[73]
*In vitro*	NPC cells	5, 10, and 15 μM	Upregulating miR-7 expression Inhibiting Skp2 expression Increasing the G2/M phase fraction	[72]
*In vivo*	CaSki-implanted nude mice	1000 and 1500 mg/kg b.w.	Downregulating VEGF, COX-2, and EGFR expression Reducing increased capillary networks Attenuating abnormalities of the capillary network pattern	[74]
*In vitro*	MCF-7 breast cancer cells	2.5 μM	Increasing Bcl-2 expression Decreasing Bax expression Inhibiting EGRF expression	[75]
*In vitro*	Patu8988 pancreatic cancer cells	10, 15, and 20 μM	Downregulating YAP and TAZ expression Inhibiting *Notch-1* expression Inducing arrest in the G_2_/M phase	[77]
*In vivo*	Albino rats with oral carcinogenesis	30 and 100 mg/kg b.w.	Decreasing the expression of PCNA, Bcl-2, SOCS1, and STAT3 Diminishing the expression of genes associated with epithelial-mesenchymal transition (EMT)	[79]
*In vitro*	MDA-MB-231 triple negative breast cancer cells	40 μM	Activating p38-MAPK Reducing the level of CDK2, CDK4, cyclin D1, and cyclin E Inducing cell cycle arrest in the G_1_/S and G_2_/M phase	[81]
*In vitro*	Ras-activated HAG-1 human adenocarcinoma cells	25 μM	Enhancing ERK1/2 Inhibiting Akt, mTOR, and S6K1 expression Inducing arrest in the G_2_/M phase	[83]
*In vivo*	Male Sprague–Dawley rats	50 mg/kg b.w.	Co-treatment with diclofenac Inhibiting the telomerase activity Upregulating the tumor suppressor proteins, p51, Rb, and p21 Inducing cell cycle arrest	[84]
*In vitro*	Lung epithelium cancer A549 cells	5 and 10 μM	Co-treatment with apigen inIncreasing p53 expression Blocking cell cycle progression in the G2/M phase	[85]
***Effects on tumor cell apoptosis***				
*In vitro*	Src-activated HAG-1 human adenocarcinoma cells	25 μM	Suppressing Bcl-xL expression Enhancing Bax expression	[83]
*In vitro*	MCF-7 breast cancer cells	50 μg/mL	Reducing *Mcl-1* gene expression Declining the viability of cells	[91]
*In vitro*	MDAH 2774, SKOV3 and PA1 human ovarian cancer cells	15 μM	Suppressing SERCA activity Disrupting Ca^2+^ homeostasis	[92]
*In vitro*	KB human oral epidermoid carcinoma cells	5 and 12.5 μM	Inhibiting the activity of ZAKα	[93]
*In vivo* & *in vitro*	DU145 human prostate cancer cells and B16 murine melanoma cells	5 μM	Curcumin analog EF24 Inhibiting miR-21 expression Enhances PTEN and PDCD4 expression Suppressing cyclin D1 and Ki67 expression	[95]
	Male NOD scid γ mice (NSG) mice	200 μg/kg b.w.		
*In vitro*	MDA-MB-231 metastatic breast and A549 lung cancer cells	10, 20 and 30 μM	Reducing the expressions of HIF 1-α and nuclear p65 (Rel A)	[97]
*In vitro*	A549 lung cancer cells	40 μM	Suppressing miR-21 expression Elevating the protein level of PTEN	[96]
*In vitro*	DLD-1, LoVo, HCT116 human colon cancer cells	12.5 µM	Co-treatment with silymarin Induced five-fold higher caspase 3/7 activity	[76]
*In vitro*	253J-Bv and T24 human bladder cancer cells	10 μM	Co-treatment with cisplatin Triggering ROS production Activating caspase 3 Upregulating p-ERK1/2 signaling	[99]
*In vitro*	Rh30 and Rh41 human alveolar rhabdomyosarcoma-derived cells	10, 25, and 50 μM	Blocking the NF-κB pathway Increasing sensitivity to ionizing radiation	[100]
*In vitro*	HCT116 human colon cancer cells	5 μM	Downregulating NF-κB activation and regulated gene products Potentiating the chemotherapy of 5-fluorouracil	[101]
*In vivo* & *in vitro*	HCT116 and HT29 human colon cancer cells Male BALB/c-nu/nu mice	10, 20, 30, and 40 μM 40 mg/kg b.w.	Downregulating NF-κB activation Inhibiting AMPK/ULK1-dependent autophagy Potentiating 5-fluorouracil-induced reduction in cells’ proliferation and invasion	[102]
***Effects on metastasis and invasion***				
*In vitro*	NPC cells	5, 10, and 15 μM	Inhibiting cell motility Suppressing invasion into the Matrigel-coated membrane	[72]
*In vitro*	Lewis lung cancer cells	20 μM	Reducing the capacity to invade through Matrigel	[105]

Akt, Protein kinase B; AMPK, AMP-activated protein kinase; Bax, Bcl-2-associated X protein; Bcl-2, B-cell lymphoma 2; Bcl-xL, B-cell lymphoma-extra large; Bik, Bcl-2-interacting killer; COX-2, Cyclooxygenase-2; EGFR, Epidermal growth factor receptor; ERK1/2, extracellular signal–regulated kinases; HNSCC, Head-neck squamous cell carcinoma; MAPK, Mitogen-activated protein kinase; mTOR, Mammalian target of rapamycin; NF-κB, Nuclear factor κB; NPC, Nasopharyngeal carcinoma; PCNA, Proliferating cell nuclear antigen; PTEN, Phosphatase and tensin homolog; ROS, Reactive oxygen species; S6K1, p70-S6 kinase 1; SERCA, Sarco/endoplasmic reticulum calcium ATPase; Skp2, S-phase kinase-associated protein 2; SOCS1, Suppressor of cytokine signaling 1; STAT3, Signal transducer and activator for 254 transcription 3; TAZ, Transcriptional coactivator with PDZ-binding motif; ULK1, Unc-51-like kinase; VEGF, Vascular endothelial growth factor; YAP, Yes-associated protein 1; ZAKα, Leucine-zipper and sterile-alpha motif kinase alpha.

**Table 2:** Hepatoprotection and related molecular mechanisms of curcumin.

Study Type	Subjects	Dose	Potential Mechanisms	Ref.
***Liver injuries induced by pollutants, drugs, and alcohol***				
*In vivo*	Swiss albino rats with CCl_4_ hepatotoxicity	8.98 μM	Maintaining cellular ROS levels Increasing the level of GR and GST Decreasing the level of NADH oxidase Increasing the activity of SDH	[112]
*In vivo*	Sprague-Dawley rats with CCl_4_ hepatotoxicity	200 mg/kg b.w.	Decreasing the activities of AST and ALT and the level of lipid peroxidase Increasing hepatic GSH content	[114]
*In vivo*	Sprague-Dawley rats with diabetes induced by streptozotocin	100 mg/kg b.w.	Decreasing hepatic endoplasmic reticulum stress marker protein and the sub-arm of unfolded protein response signaling protein Inhibiting TNF-α, IL1β, phospho-p38 MAPK, and ASK1 in liver tissues	[116]
*In vivo*	CD1 mice with paracetamol hepatotoxicity	35, 50, and 100 mg/kg b.w.	Attenuating the decrease in oxygen consumption in the membrane potential, ATP synthesis, aconitase activity, and activity of respiratory complexes, I, III, and IV	[117]
*In vivo*	Kunming mice with alcoholic fatty liver	60 mg/kg b.w.	Suppressing ethanol-induced pathways, including biosynthesis of unsaturated fatty acids, fatty acid biosynthesis, and pentose and glucuronate interconversions Inhibited glyoxylate, dicarboxylate, and pyruvate metabolism	[118]
*In vivo*	Male ICR mice with alcoholic fatty liver	20 μM	Attenuating hepatocyte necroptosis Increasing hepatic Nrf2 expression	[119]
***Nonalcoholic fatty liver disease***				
*In vivo*	Peripheral blood mononuclear cells	10 μM	Reducing cytoplasmic translocation of HMGB1, protein expression of TLR4, and nuclear translocation of NF-κB Suppressing glypican-3 expression, VEGF, and pro-thrombin in NASH liver	[124]
	C57BL/6J mice with NASH-hepatocellular carcinoma	100 mg/kg b.w.		
*In vivo* & *in vitro*	C57BL/6J mice with NAFLD	2 g curcumin/kg of diet	Preventing high-fat diet-induced liver injury, metabolic alterations, and intrahepatic CD4^+^ cell accumulation Reducing the pro-inflammatory and pro-oxidant effects on liver macrophages.	[122]
*In vivo*	TRPM2 knockout Hooded Wistar rats	5 μM	Inhibiting the activation of TRPM2 channels Restoring Ca^2+^ levels Reducing oxidative stress Lowering the risk of NASH	[124]
***Liver fibrosis***				
*In vivo*	Sprague-Dawley rats with alcohol-induced hepatic fibrosis	50 μM	Inhibiting HSCs proliferation Stimulating endoplasmic reticulum stress Suppressing the TGF-β/Smad signaling pathway Reducing the viability of HSCs	[125]
*In vivo* & *in vitro*	Sprague-Dawley rats with CCl_4_-induced hepatic fibrosis	100, 200, and 400 mg/kg b.w.	Reducing extracellular matrix overproduction in HSCs Downregulating CBR type 1 Upregulating CBR type 2	[127]
	HSCs isolated from rats	10, 20, and 30 μM		
*In vivo* & *in vitro*	Sprague-Dawley rats with CCl_4_-induced hepatic fibrosis	100, 200, and 400 mg/kg b.w.	Disrupted PDGF-R/ERK and mTOR pathways Activating PPAR-γ Reducing the angiogenic properties of HSCs	[128]
	HSCs isolated from rats	20 μM		
*In vivo* & *in vitro*	Sprague Dawley rats with CCl_4_-induced hepatic fibrosis	200 mg/kg b.w.	Upregulating miR-29b expression Downregulating DNA methyltransferase 3b Upregulating PTEN Inhibiting activated HSCs	[129]
	Rat HSC-T6 cells	20 μM		

ALT, Alanine transaminase; ASK1, Apoptosis signal-regulating kinase 1; AST, Aspartate aminotransferase; CBR, Cannabinoid receptors; CCl_4_, Carbon tetrachloride; CD4, Cluster of differentiation 4; ERK, Extracellular signal-regulated kinases; GR, Glutathione reductase; GSH, Glutathione; GST, Glutathione S-transferases; HMGB1, High mobility group box 1; HSCs, Hepatic stellate cells; ICR, Institute of Cancer Research; IL1β, Interleukin 1β; MAPK, Mitogen-activated protein kinase; mTOR, Mammalian target of rapamycin; NADH, Nicotinamide adenine dinucleotide; NAFLD, Nonalcoholic fatty liver disease; NASH, Non-alcoholic steatohepatitis; NF-κB, Nuclear factor κB; Nrf2, Nuclear factor (erythroid-derived 2)-like 2; PDGF-R, Platelet-derived growth factor- receptor; PTEN, Phosphatase and tensin homolog; ROS, Reactive oxygen species; TGF-β, Transforming growth factor-beta; TLR4, Toll-like receptor 4; TNF-α, Tumor necrosis factor-α; TRPM2, Transient receptor potential melastatin 2; SDH, Succinate dehydrogenase; VEGF, vascular endothelial growth factor.
